# Genome-wide DNA methylation analysis in obese women predicts an epigenetic signature for future endometrial cancer

**DOI:** 10.1038/s41598-019-42840-4

**Published:** 2019-04-23

**Authors:** Masaru Nagashima, Naofumi Miwa, Hajime Hirasawa, Yukiko Katagiri, Ken Takamatsu, Mineto Morita

**Affiliations:** 1 Department of Obstetrics and Gynecology, Graduate School of Medicine, Toho University, 6-11-1 Omori-nishi, Ota-ku, Tokyo 143-8541 Japan; 2 Department of Physiology, Graduate School of Medicine, Toho University, 5-21-16 Omori-nishi, Ota-ku, Tokyo 143-8540 Japan; 3 Department of Physiology, Saitama Medical University, 38 Moro-hongo, Moroyama, Iruma-gun, Saitama, 350-0495 Japan

**Keywords:** Cancer epigenetics, Cancer epigenetics

## Abstract

Aberrant DNA methylation is associated with the oncogenesis of a variety of human cancers, including endometrial cancer (EC), the seventh most common cancer among women. Obesity is known to be a high-risk factor for EC; however, whether obesity influences DNA methylation in the presymptomatic uterus and if this influences EC development remain unclear. Here, we performed genome-wide DNA methylation analysis of isolated endometrial epithelial cells obtained from obese presymptomatic participants. Using the Illumina MethylationEPIC array (850 K), we identified 592 differentially methylated regions (DMRs), most of which undergo hypomethylated changes. These DMRs were enriched for pyrimidine metabolism, Epstein-Barr virus infection, and B cell signaling pathways, indicating obesity-related dysregulation of certain metabolic processes in the presymptomatic uterus. Comparison of the DMRs with those in stage I EC revealed that 54 DMRs overlapped; additionally, B cell signaling and Epstein-Barr virus infection pathways were shared between the presymptomatic uterus of obese women and stage I EC with greater hypomethylation in women with EC than in presymptomatic obese women. These findings indicated that obesity influences DNA methylation in presymptomatic endometrial epithelial cells, and persistent dysregulation of DNA methylation in obese women may result in EC development.

## Introduction

Endometrial cancer (EC) is the seventh most common cancer among women, with 13,951 new cases and 2,243 deaths in Japan in 2014^1^. EC originates from endometrial epithelial cells, whereas endometriosis develops from endometrial stromal cells. The disease rate increases with age, peaking in the fifth-sixth decades of life. Based on differences in epidemiology and histopathology, there are two subtypes of EC: type I and type II. Type I endometrioid endometrial cancer accounts for approximately 80% of cases, occurs frequently in pre-and peri-menopausal women, is often well differentiated, and is correlated with obesity and overexposure to estrogen. Type II non-endometrioid endometrial cancer is more common in older, non-obese post-menopausal women and is poorly differentiated. Recent evidence revealed genetic alterations in the RTK/RAS/β-catenin and PI3K/AKT/PTEN pathways in endometrial tumor tissue^2^; however, it has also been reported that a significant subset (*e.g*. ~50%)^3^ of EC does not contain genetic mutations in PTEN, TP53, or CTNNB1, suggesting an etiology other than common genetic mutations. One of the biological processes frequently contributing to oncogenesis is epigenetic modification: heritable changes in gene expression without mutation of the amino acid sequence. A well-known epigenetic event is DNA methylation. DNA methylation regulates gene expression and microRNA stability, contributes to the cellular phenotype, and plays important roles in various cellular processes, including development, genetic imprinting, immune response, and aging^4–6^.

In DNA methylation, a methyl group (-CH_3_) is covalently bound to the cytosine residues of CpG dinucleotides, forming 5-methylcytosine (mCpG), which is mediated by several types of DNA cytosine methyltransferases. CpG dinucleotides are frequently localized in clusters in/around gene promoter regions and control gene expression by regulating promoter activity. These clusters are known as CpG islands, and generally harbor a CpG content of 60–70%. Additionally, the methylation of CpG also takes place in the 5′-upstream and 3′-downstream regions of CpG islands, known as N-, S-shores, and N-, S-shelves within the gene body and in intergenic chromatin regions (called OpenSea) (for an overview of CpGs, see refs^7,8^). Aberrant CpG methylation has been observed in a variety of diseases such as diabetes, cardiovascular diseases and cancer^9,10^; the average methylation level of CpG sites in abnormal cells is sometimes increased (hypermethylation) or decreased (hypomethylation) compared to in normal cells. Such methylation changes have been observed in endometrial cancer^2,11^. DNA methylation is also considered as a plastic/reversible cellular process. For example, obesity-related alterations in methylation in human skeletal muscle reverts to the normal methylation pattern after Roux-en-Y bypass, a treatment for obesity, indicating that DNA methylation depends on environmental risk factors^12^. Several risk factors for EC have been known, including age, obesity, diabetes, and postmenopausal hormone therapy; for example, obesity increases the rate of EC occurrence (risk ratio, 1.59)^13^. This epidemiology raises the intriguing hypothesis that high-risk factors induce aberrant DNA methylation, and when the factor is sustained, persistent dysregulation of DNA methylation may cause diseases. If this is the case, aberrant DNA methylation would be observed even when the disease-related symptoms have not yet emerged.

To explore this hypothesis, we prepared genomic DNA from isolated endometrial epithelial cells obtained from obese women, performed epigenome-wide association analysis (EWAS) using the Illumina MethylationEPIC array that covers >850 K of CpG sites, and identified differentially methylated regions (DMRs). Next, to examine whether the observed alterations remain in early-stage EC, we extracted DMRs in the tissue of early-stage EC using a publicly available database, and identified DMRs that overlapped between obese women and early-stage EC. Our study design is summarized in Supplementary Fig. S1. This is the first study to use specimens of isolated (*i.e*., not mixture of different types of cells) human endometrial epithelial cells from presymptomatic obese women for EWAS. This analysis provides key molecular insights into the pathogenesis of EC through obesity-related dysregulation of epigenetic modification, and may be useful for developing disease-related biomarker(s) and/or drug-oriented compounds for preventing EC.

## Materials and Methods

### Tissue collection

Tissue collection was approved by the Ethics Committee of Toho University (#26087). Written informed consent was obtained from all participants who were Japanese women ranging from 25- to 45-year-old. The study participants included five normal-weight (body mass index; BMI < 25) and three obese (BMI > 30) participants. They were diagnosed with endometrial hyperplasia in the past but had normal endometrial epithelial cells according to cytological examination at the time of our study (Supplementary Table S1). Notably, no participants showed hyperplasia with atypia. Furthermore, the participants were not basically treated with any medication. All methods were performed in accordance with the relevant guidelines and regulations.

### Cell culture of human endometrial epithelial cells

Human endometrial epithelial cells were isolated from the excised endometrial tissue comprised of different types of cells according to the previous report^14,15^. Briefly, the excised endometrial tissues were minced and digested in 50 mL of 0.25% collagenase (Sigma, St. Louis, MO) and 0.005% deoxyribonuclease (Sigma) solution in DMEM/F12 medium at 37 °C for 90 min. After rinse, the tissue was triturated in DMEM/F12, and the cell suspension was successively filtered through a series of filter (250-, 100-, and 37-μm mesh size, Fisher Scientific, Waltham, MA) to remove endometrial stromal cells. Isolated endometrial epithelial cells were retained on the upper side of the 37-μm sieve and plated at a density of 0.5 × 10^6^ cells/cm^2^ onto two plastic culture plates, one of which was used for DNA preparation and the other for immunohistochemical analysis to determine the ratio of epithelial cells. Cells were maintained in DMEM medium containing 10% fetal calf serum, 30 ng/mL endothelial cell growth factor, and Pen/Strep, and the medium was exchanged twice per week.

### Antibodies

Anti-cytokeratin 18 (CK18, Cell Signaling Technology, Beverly, MA) and anti-vimentin (Santa Cruz Biotechnology, Santa Cruz, CA) antibodies were used to detect endometrial epithelial cells, and endometrial stromal cells, respectively.

### Immunohistochemistry

Cultured endometrial epithelial cells were fixed in 4% paraformaldehyde for 5 min at room temperature. After permeabilization and blocking with 5% normal goat serum, the cells were treated simultaneously with two antibodies (anti-CK18 antibody,1:100; anti-vimentin antibody, 1:100) at 4 °C overnight. After rinse, the specimens were treated with secondary antibodies (Alexa Fluor 594 and Alexa Fluor 488; 1:1000, Molecular Probes, Invitrogen, Carlsbad, CA) and nuclei-stained by DAPI (1 µg/mL; Dojindo, Kumamoto, Japan). Stained specimens were observed using either a conventional fluorescence microscope (Olympus, Tokyo, Japan) or inverted confocal laser microscope (LSM510; Carl Zeiss, Oberkochen, Germany) with an appropriate set of excitation and emission filters. The samples in which the population ratio of CK18+ cells (the number of CK18+ cell/the number of total cells) was >80% were used for subsequent DNA methylation analysis (Supplementary Table S1).

### DNA methylation analysis

Cultured epithelial cells were recovered at ~21 days *in vitro* (DIV21) and genomic DNA was prepared according to a standard protocol (NucleoSpin, Takara Bio, Shiga, Japan). Genomic DNA (~1 μg) was bisulfite-treated using a DNA methylation kit (Takara Bio), and DNA methylation was analyzed using an Infinium MethylationEPIC array, with all other procedures conducted according to the standard Infinium HD array methylation protocol (Illumina, San Diego, CA, USA). This array contains >850,000 CpG sites covering regulatory genomic regions, including enhancers, transcription factor binding sites, and open chromatin regions^16^. Raw methylation data were quality-checked based on multiple steps built for control probes: staining, hybridization, extension, specificity, and subsequent statistical analysis was conducted using R (v3.1.1), and GenomeStudio Methylation Module (v1.8) available on GenomeStudio (v2011.1). Individual probes were then filtered based on the Illumina detection *p*-value and all CpG sites with a mean p < 0.05, [Abs(delta) >0.2 (*i.e*., >20% change of β-value) or LogRatio >0.6 (*i.e*., >60% change in the logarithm of the ratio of β-values)] were considered detected and used for subsequent analysis. The DMRs were annotated using UCSC RefSeq tracks. DMRs in stage I EC were extracted from the National Cancer Institute portal site (GDC portal, https://portal.gdc.cancer.gov/). Probe information with signal data were converted and imported into GenomeStudio. The DMRs of obese vs normal-weight individuals and those of stage I EC were compared using GenomeStudio, and matched RefGenes were identified.

### Gene ontology and pathway analysis

Gene ontology (GO) term and pathway analyses for RefSeqs were conducted using the DAVID functional annotation bioinformatics (DAVID Bioinformatics Resources v6.8; https://david.ncifcrf.gov)^17^, with reference to databases: the KEGG (https://www.genome.jp/kegg/kegg1.html) and the REACTOME (https://reactome.org).

## Results

### Morphological and immunological features of isolated endometrial epithelial cells

Cultured endometrial epithelial cells formed densely packed colonies with a polyhedral shape and showed a robust reaction with anti-CK18 antibody, but no reaction with anti-vimentin antibody (Supplementary Fig. S2). In contrast, endometrial stromal cells showed a fibroblast-like elongated and more granular shape and reacted with anti-vimentin antibody, but not with anti-CK18 antibody (Supplementary Fig. S3).

### Overall methylation profile of endometrial epithelial cells of obese cases

**Figure 1:**
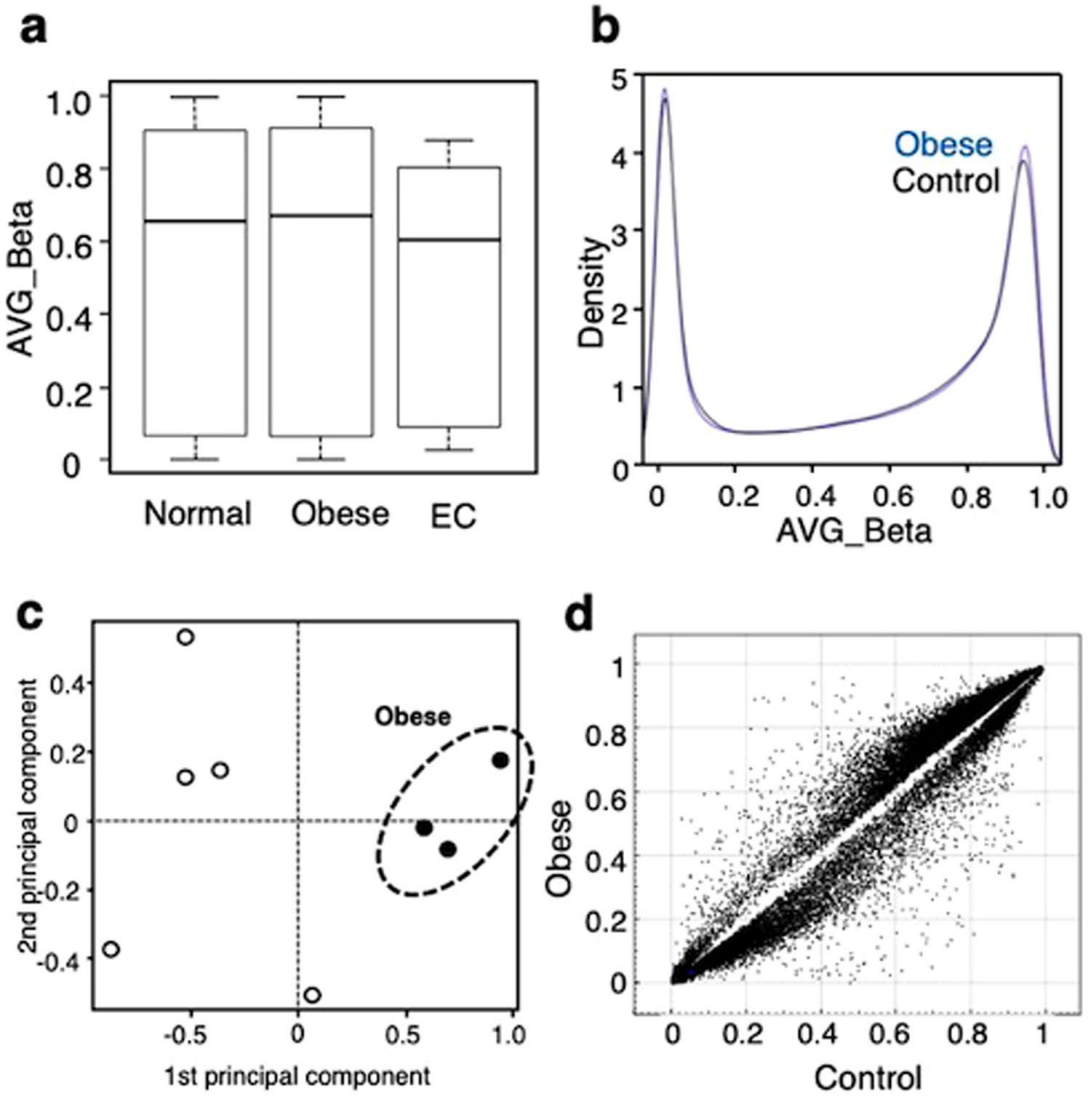
Overall CpG methylation pattern in obese and control women. (**a)** Box plot of the methylation profile of endometrial epithelial cells from normal (n = 5) and obese (n = 3) women, together with the profile of stage I endometrial cancer (n = 41). (**b)** Density plot of the averaged β-values of CpG sites of obese (blue) and normal women (black). (**c)** Principal component analysis revealed a region for the averaged β-values of CpG sites of obese women (closed circle, dashed line) marked distinctly from those of normal women (open circle). (**d)** Scatter plot comparing the averaged β-values for individual CpGs between normal and obese cases. Plotted data represent CpG sites that differed in the averaged β-value by at least 0.2.

A box plot revealed a non-significant difference between obese women (BMI > 30, hereafter referred to as cases) and normal weight women (BMI < 25, hereafter referred to as controls) (Fig. 1a,b). A density plot showed a bimodal methylation pattern with two peaks at lower and higher β-average ranges, which was similar between cases and controls. The results shown in these two plots suggest that obesity without obstetrical symptoms does not significantly affect the overall methylation profile. In contrast, tissue from the early stage of EC (stage I) showed a reduced methylation pattern in the box plot compared to cases and controls (Fig. 1a), indicating a profound change in the overall methylation profile in stage I of EC. Principal component (PC) analysis revealed that cases and controls were distinctly grouped (Fig. 1c, cases surrounded by dashed line). Additionally, a Scatchard plot revealed that a significant portion (>0.5% of total probes) of probes had different β-average values when we set the cutoff delta value to 0.2 (*i.e*., >20% change in β-value) between cases and controls (Fig. 1d). These two (PC and Scatchard) analyses indicated significant alterations in the methylation pattern in endometrial epithelial cells from cases compared to those from controls.

### Characterization of differentially methylated regions

**Figure 2:**
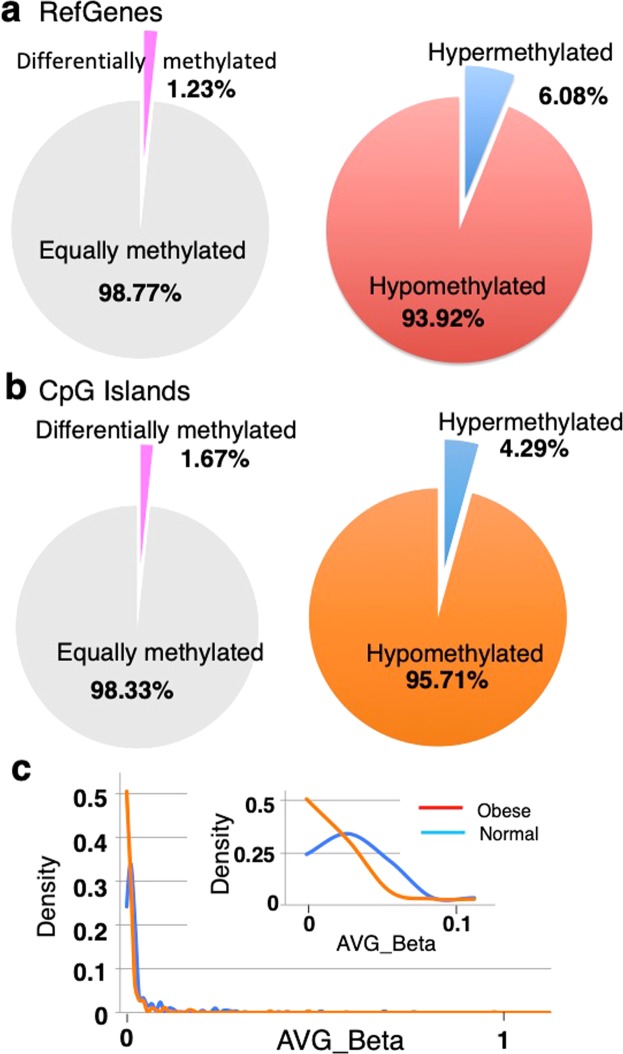
Distribution of hypo- and hyper-methylated CpG sites. **(a)** The ratio of hypo- and hyper-methylation of differentially methylated RefGenes. (**b)** The ratio of hypo- and hyper-methylation of differentially methylated CpG contexts. (**c)** Fitted histogram of the averaged β-values of CpG sites of obese (red) and normal women (blue). Frequency from 0 to 1 for the averaged β-values were plotted and fitted with the window value of 0.01. Inset: Fitted histogram enlarged the region within 0–0.1 for the averaged β-values.

Our analysis found 10,601 differentially methylated probes (DMPs) among 862,427 CpG probes, and the ratio of DMPs/total probes was ~1.2% (p < 0.05, delta >0.2)(data not shown). Among these DMPs, most exhibited a hypomethylated pattern in cases (~94% for RefGenes, ~96% for CpG sites; Fig. 2a,b), while a small number (~1.2% for RefGenes, ~1.6% for CpG sites) exhibited a hypermethylated pattern. To investigate whether this preference of hypomethylated change (>90% of total changes) occurred within a specific β-average range or in the entire region, we compared the methylation levels of DMPs between cases and controls and found that hypomethylated changes in DMPs were pronounced in lower β-average value ranges (Fig. 2c), suggesting that methylation sites at the low β-average range are more susceptible to demethylation processes associated with obesity. Among the genomic elements (*i.e*., promoter, exon, intron, intergenic), the distribution pattern of DMPs was almost similar to that of the original distribution of CpG probes in the MethylationEPIC array (Fig. 2c in 16). An exceptional distribution was observed in the intergenic region [63% (hypomethylated) and 74% (hypermethylated), greater than 33% of the original distribution](Supplementary Fig. S4). The distribution pattern of DMPs in the CpG contents was also similar to that of the original distribution of CpG probes in the array, except for the distribution ratios in the shore regions (*e.g*., 23%, summation of 12 for N Shore and 11% for C Shore, hypomethylated; Supplementary Fig. S4b), which were greater than that of the original (11%) (Fig. 2c in 16).

### Characterization of differentially methylation in RefGenes promoter regions

**Figure 3:**
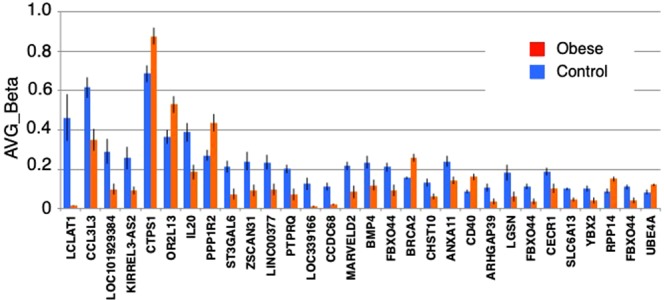
Difference of the averaged of β-values of RefGenes. Difference of the averaged β-values of top 30 RefGenes of DMRs between obese (red) and control (blue).

To correlate DMPs with genetic loci, we focused on differentially methylated RefGene promoter regions (DMRs) among the DMPs, and found 592 DMRs (p < 0.05). Table 1 shows the top 30 representative DMRs, together with the difference in β-values between cases and controls. The methylation levels in 23 of 30 DMRs were decreased significantly (Table 1). The largest decrease in methylation level was observed for LCLAT1 (lysocardiolipin acyltransferase1, ~97% decrease), followed by CCL3L3 (C-C motif chemokine ligand3 like 3, 44% decrease) (Fig. 3). Annotation by our Gene Ontology (GO) analysis showed that DMRs were enriched for cellular functions including chromosome, mitotic spindle, polyubiquitination, acetyl-CoA biosynthetic process, apoptotic signaling, and NIK/NF-kappaB signaling (Supplementary Table S2), suggesting that obesity affects certain cellular events in the presymptomatic uterus.

**Table 1:** RefGenes of differentially methylated regions (DMRs) with greatest difference in the averaged β-values.

GENE-SYMBOL	AVE_Beta of normal group	AVE_Beta of obese group	Delta	RefSeq_ACCESSION	ENTREZ_GENEID	TYPE_OF_GENE	DESCRIPTION
LCLAT1	0.4609843	0.0145585	−0.526158	NM_001304445	253558	protein-coding	lysocardiolipin acyltransferase 1
CCL3L3	0.6155401	0.3475144	−0.25072	NM_001001437	414062	protein-coding	C-C motif chemokine ligand 3 like 3
LOC101929384	0.289783	0.09906	−0.23547	NR_120475	101929384	ncRNA	uncharacterized LOC101929384
KIRREL3-AS2	0.2568615	0.0921788	−0.208665	NR_046986	100874251	ncRNA	KIRREL3 antisense RNA 2
CTPS1	0.6850519	0.87511	0.202434	NM_001301237	1503	protein-coding	CTP synthase 1
OR2L13	0.3626859	0.5284353	0.1977	NM_175911	284521	protein-coding	olfactory receptor family 2 subfamily L member 13
IL20	0.3886124	0.1850412	−0.197166	NM_018724	50604	protein-coding	interleukin 20
PPP1R2	0.2695188	0.4360325	0.156859	NM_001291505	5504	protein-coding	protein phosphatase 1 regulatory inhibitor subunit 2
ST3GAL6	0.213688	0.0722118	−0.14837	NM_001271142	10402	protein-coding	ST3 beta-galactoside alpha-2,3-sialyltransferase 6
ZSCAN31	0.24028	0.0924391	−0.132292	NM_001243244	64288	protein-coding	zinc finger and SCAN domain containing 31
LINC00377	0.2347052	0.0984455	−0.129794	NR_125770	103724386	ncRNA	long intergenic non-protein coding RNA 377
PTPRQ	0.2012982	0.0693708	−0.129505	NM_001145026	374462	protein-coding	protein tyrosine phosphatase, receptor type Q
LOC339166	0.1262455	0.0104135	−0.125039	NR_040000	339166	ncRNA	uncharacterized LOC339166
CCDC68	0.1115733	0.0209919	−0.122825	NM_025214	80323	protein-coding	coiled-coil domain containing 68
MARVELD2	0.2159227	0.0842055	−0.118099	NM_001038603	153562	protein-coding	MARVEL domain containing 2
BMP4	0.23403	0.1188948	−0.11401	NM_001202	652	protein-coding	bone morphogenetic protein 4
FBXO44	0.211308	0.091532	−0.111535	NM_183412	93611	protein-coding	F-box protein 44
BRCA2	0.156211	0.2569725	0.103704	NM_000059	675	protein-coding	breast cancer 2
CHST10	0.1315424	0.0611276	−0.09368	NM_004854	9486	protein-coding	carbohydrate sulfotransferase 10
ANXA11	0.2397733	0.1431341	−0.090493	NM_001278407	311	protein-coding	annexin A11
CD40	0.086086	0.1608967	0.08881	NM_001250	958	protein-coding	CD40 molecule
ARHGAP39	0.1064887	0.0375751	−0.078823	NM_001308208	80728	protein-coding	Rho GTPase activating protein 39
LGSN	0.1821024	0.061384	−0.077074	NM_016571	51557	protein-coding	lengsin, lens protein with glutamine synthetase domain
FBXO44	0.1132692	0.0343917	−0.076007	NM_001304791	93611	protein-coding	F-box protein 44
CECR1	0.1888989	0.1022042	−0.074676	NM_177405	51816	protein-coding	cat eye syndrome chromosome region, candidate 1
SLC6A13	0.1002082	0.0445989	−0.069311	NM_001190997	6540	protein-coding	solute carrier family 6 member 13
YBX2	0.102823	0.0424958	−0.060535	NM_015982	51087	protein-coding	Y-box binding protein 2
RPP14	0.0891559	0.1503356	0.056264	NR_049755	11102	protein-coding	ribonuclease P/MRP 14kDa subunit
FBXO44	0.1109388	0.0407703	−0.054111	NM_183413	93611	protein-coding	F-box protein 44
UBE4A	0.0837289	0.1201833	0.052674	NM_004788	9354	protein-coding	ubiquitination factor E4A

Top 30 RefGenes with greatest difference in the averaged β-values were listed. Top 30 RefGenes Promoter Regions.

### Pathway analysis of DMRs

To specify biologically relevant pathways among the obesity-associated methylation cellular events, we performed the KEGG pathway analysis using DMRs, and found that DMRs were significantly enriched in three pathways (p < 0.05): (i) pyrimidine metabolism, (ii) Epstein-Barr (EB) virus infection, and (iii) B cell signaling pathways (Supplementary Fig. S5). Similar to the overall characterization of DMRs, the methylation levels in many genes decreased (*i.e*., 9 of 11 genes were hypomethylated, whereas the rest were hypermethylated). In the pyrimidine metabolism pathway, the methylation levels of UCKL1 (uridine-cytidine kinase 1) and CTPS1 (CTP synthase 1) increased, while those of POLR3A (RNA polymerase III), RRM2B (ribonucleoside-diphosphate reductase subunit M2 B) and DUT (deoxyuridine triphosphatase) decreased in the cases. In the EB virus infection pathway, POLR3A and HDAC1 (histone deacetylase 1) showed a decrease in methylation levels; and in the B cell signaling pathway, ATF2 (activating transcription factor 2), IL10R (interleukin 10 receptor subunit alpha), NFkappaB2 and EIF2AK4 (eukaryotic translation initiation factor 2 alpha kinase 4) showed decreased methylation. Thus, obesity may induce dysregulation of these signaling pathways, rendering the endometrial epithelial cells susceptible to disease development (see Discussion). We also performed pathway analysis using another database (REACTOME), and found that HDAC1 is shared between both databases (KEGG and REACTOME), suggesting obesity-associated dysregulation of HDAC1-related signaling pathways (data not shown).

### Overlap of DMRs between obese cases and early stage of endometrial cancer

**Figure 4:**
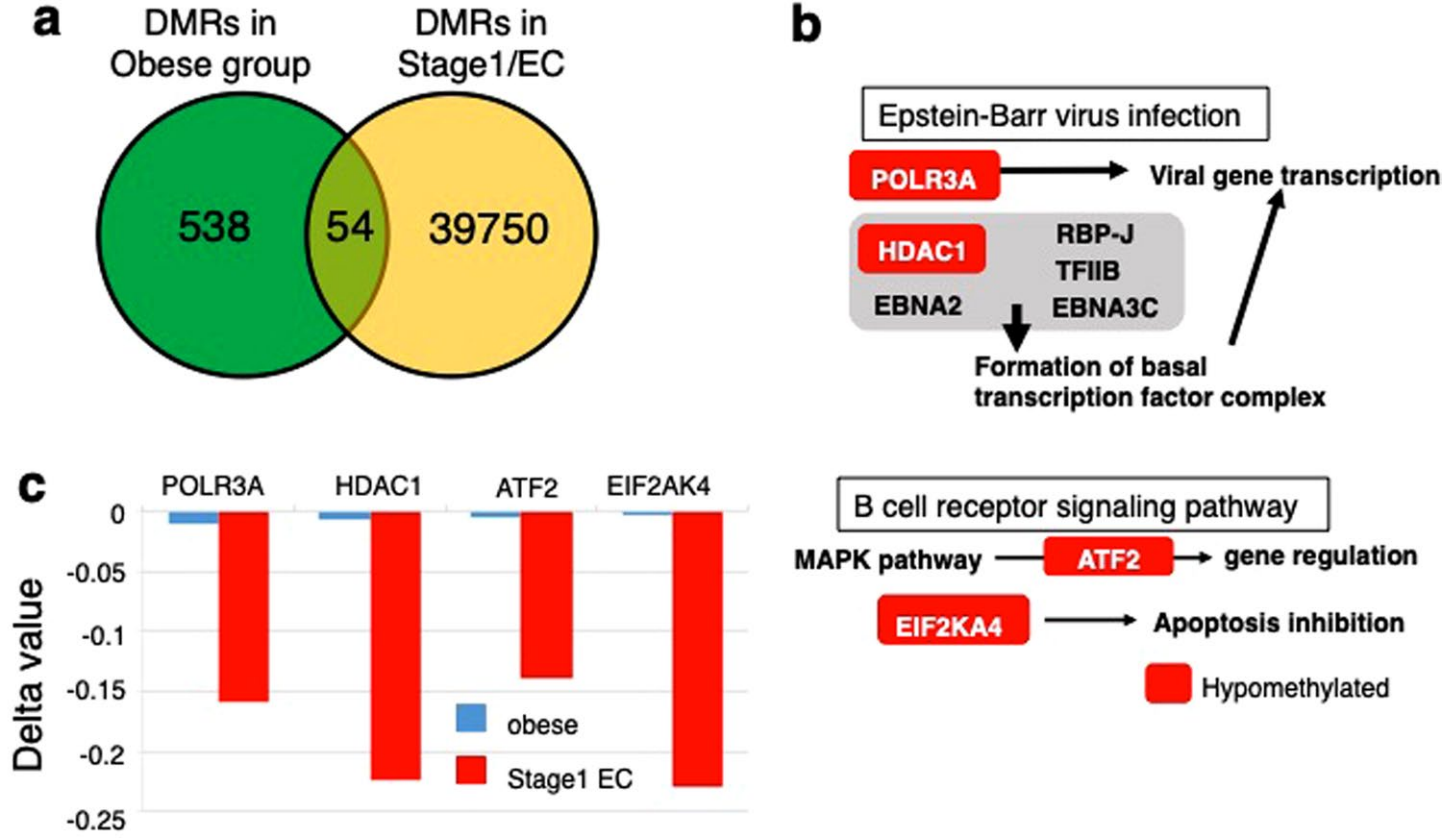
Overlap of differentially methylated regions (DMRs) between the obese cases and stage I endometrial cancer. (**a)** Venn graph showing the overlap of DMRs that were identified in our study and those of stage I endometrial cancer. (**b)** Shared DMRs are enriched for EB virus and B cell signaling pathways, and the methylation levels of all shared genes are decreased (red) in two pathways. (**c)** The extent of the decrease in methylation levels in shared DMRs. The extent of decrease in methylation levels in shared DMRs were greater in stage I EC, compared with those of obese women.

We considered that progression of aberrant methylation in persistent obesity may lead to EC occurrence. If so, the responsible gene(s) should, at least, overlap between the presymptomatic obese cases and samples from early stage EC. To examine this potential overlap of DMRs, we first extracted DMRs (total of 39,804 RefGenes) in stage I EC from a publicly available database (n = 41, n = 26 for stage I, and normal, respectively; p < 10^−9^, the value below the Bonferroni corrected one), and found that 54 genes overlapped with DMRs in our study (Fig. 4a). The methylation levels of overlapped DMRs were often low, indicating that obesity-associated changes preferentially occurred in CpGs whose methylation levels were low. GO analysis revealed that the overlapped DMRs were enriched in genes involved in chromatin binding, transcription factor binding, and neural development (p < 0.05) (Supplementary Table S3). Furthermore, we carried out functional pathway analysis to investigate the potential shared metabolic processes in obesity and EC pathogenesis using 54 overlapping DMRs. KEGG pathway analysis showed overlap of POLR3A and HDAC1 in the EB virus infection pathway, and ATF2 and EIF2AK4 in the B cell signaling pathway (Fig. 4c and Supplementary Table S4). All four of these shared DMRs were hypomethylated both in cases and the stage I of EC; furthermore, the degree of hypomethylation was greater in the stage I of EC compared to that in cases, suggesting that an enhanced level of hypomethylation is correlated with EC occurrence (for a proposed model, see Supplementary Fig. S6).

## Discussions

Obesity is associated with active but low-grade inflammation, and recent EWAS identified a set of candidate genes that couple obesity and tissue malfunction through epigenetic dysregulation of gene expression^12,18–20^, although the molecular mechanism whereby obesity induces dysregulation of DNA methylation has not been elucidated. The set of candidate genes comprise >180 genes in blood cells, >120 genes^21^ and >700 genes in adipose tissue^20^, which confirms that lifestyle- and environmental-factors regulate global epigenetic modification, affecting a wide variety of cellular processes such as immune/inflammatory signaling, stress response, vesicle transport, apoptosis, and cAMP signaling. Consistent with prior research using other tissues, our study revealed that obesity induces substantial methylation changes (mostly hypomethylation) in endometrial epithelial cells: to our knowledge, this is the first study to perform EWAS using such cells. We compared DMRs identified in our present study and those previously reported, and found that GCA (grancalcin)^22^ and MAD1L1 (mitotic arrest deficient 1 like 1)^23^ overlapped in our study. This low amount of overlap (2 of 592) between our study and previously reported research may be due to the difference in the DNA source: endometrial epithelial cells in our study, as compared with peripheral blood cells, and/or adipose tissues in the past studies. The observed differences may be correlated with tissue-specific patterns of epigenetic regulation.

Our pathway analysis revealed several genes in three pathways in which DMRs were enriched: (i) pyrimidine metabolism, (ii) B cell signaling, and (iii) EB virus infection pathways (Supplementary Fig. S5). Pyrimidine metabolism is known to be influenced by obesity^24,25^, and epigenetic modification of pyrimidine metabolism may affect the cell cycle, differentiation, and proliferation through regulation of DNA and RNA metabolism, including non-coding RNAs and microRNAs^24^. The B cell signaling pathways is also known to be involved in obesity. In obese mice, chemokines secreted from adipocytes stimulate pro-inflammatory B cells, impairing B cell function^26^. This is the first study to demonstrate the potential dysregulation of the EB virus infection pathway in obesity. Notably, nearly all adult women in Japan and USA have experienced a previous EB virus infection: >90% of adult women are seropositive for the EB virus^26,27^. This suggests that participants in our study were likely infected with the EB virus, and EB virus signaling in endometrial epithelial cells was modulated in an epigenetic manner in obesity. Thus, obesity appears to regulate global epigenetic modification, particularly affecting three pathways and allowing the tissue to develop obesity-related comorbidities, including cancer.

We extracted DMRs in stage I EC from a publicly available database, compared these with the DMRs in our study, and found 54 shared DMRs (Fig. 4a). Our pathway analysis of 54 DMRs revealed enrichment in two pathways: (i) EB virus infection and (ii) B cell signaling pathways (Fig. 4b). Recent studies showed that EB-virus infection is involved in gastric oncogenesis through epigenetic modification of intracellular signaling in gastric epithelial cells^28,29^, although the underlying molecular mechanism remains unclear. B cell signaling is known to be regulated by numerous transcription factors, including hypoxia-inducible factor (HIF), the methylation level of which is associated with obesity^18^. Alterations in HIF methylation may lead to changes in its expression level, impairing B cell signaling to cause oncogenesis of EC. Notably, epigenetic repression of connexin 43, a gap junction protein, contributes to obesity-associated EC development^30^.

Decreased methylation in four RefGenes (POLR3A and HDAC1 in the EB virus infection pathway, ATF2 and EIF2AK4 in the B cell signaling) was enhanced in stage I EC (Fig. 4c) compared to in the healthy but high-BMI participants in our study. Interestingly, this suggests that persistent obesity may exaggerate epigenetic dysregulation and hamper these signaling, contributing to the oncogenesis of EC (see model in Supplementary Fig. S6). If so, renormalization of DNA methylation of the genes whose methylation has been changed in obesity would be absolutely critical for preventing EC oncogenesis, because the methylation is considered as a plastic/reversible cellular process (see Introduction). Unfortunately, this work does not address the molecular mechanism of dysregulation of DNA methylation that leads to EC oncogenesis. Human obesity-related gene expression omnibus data for endometrial epithelial cells are not yet publicly available; therefore, we could not validate the effects on expression of the identified gene.

Future study is needed to examine whether changes in the DNA methylation level induces the expression of the genes detected in this study. Additionally, comparison of CpG sites of shared RefGenes (*i.e*., POLR3A and HDAC1 in EB virus infection pathway; ATF2 and EIF2AK4 in B cell signaling) and the location of single-nucleotide polymorphisms (SNPs) showed that none of the four DMRs were condensed within SNP clusters (Supplementary Fig. S7), suggesting that changes in methylation array signals are unlikely due to DNA sequence variation, but rather to altered methylation. It is, however, necessary to verify which CpG sites are most correlated with the oncogenesis of EC.

In conclusion, we identified substantial alterations in DNA methylation in endometrial epithelial cells in obesity, and found enrichment of the DMRs in several pathways. In addition, we showed that this altered methylation in the DMRs is shared with stage I (early stage) of endometrial cancer, and the degree of hypomethylation is greater in EC. These findings suggest that obesity causes dysregulation of methylation, exerting detrimental effects on certain metabolic pathways, further contributing to the oncogenesis of EC. Our study also reveals the potential of these DMRs to function as biomarkers for the development of EC and to be targets for novel drug development for cancer prevention.

## Supplementary information

Supplementary Information

## Data Availability

Our DNA methylation data have been submitted to the NCBI Gene Expression Omnibus under Accession Number GSE122148.
