# Supplementary material for: Genome-wide DNA methylation analysis in obese women predicts an epigenetic signature for future endometrial cancer
Source: Sci Rep. 2019 Apr 23;9:6469. doi: 10.1038/s41598-019-42840-4 (PMC6478742; doi:10.1038/s41598-019-42840-4)
Supplement: Supplementary file 1 — Supplementary Information [file 41598_2019_42840_MOESM1_ESM.pdf]

Supplementary Information

Genome-wide DNA methylation analysis in obese women predicts an epigenetic signature for future endometrial cancer.

Masaru Nagashima[1], Naofumi Miwa[2,3*], Hajime Hirasawa[3], Yukiko Katagiri[1*], Ken Takamatsu[2], Mineto Morita[1]

[1]Department of Obstetrics and Gynecology, Graduate School of Medicine, Toho University, 6-11-1 Omori-nishi, Ota-ku, Tokyo 143-8541
[2]Department of Physiology, Graduate School of Medicine, Toho University, 5-21-16 Omori-nishi, Ota-ku, Tokyo 143-8540,
[3]Department of Physiology, Saitama Medical University, 38 Moro-hongo, Moroyama-machi, Iruma-gun, Saitama 350-0495

Figure Legends

Supplementary Fig. S1 Summarized study design.
Overview of the flow in the present study.

Supplementary Table S1 Case characteristics of normal-weight and obese participants
Characteristics of participants (*i.e.*, Age. BMI, gravidity, parity, day of menstrual cycle, Purity of endometrial epithelial cells, others) were listed.

Supplementary Fig. S2 Preparation of human endometrial epithelial cells.
Human endometrial epithelial cells were isolated from the uteri of the participants, and cultured for ~21 days. Isolated endometrial epithelial cells were fixed and double-stained with anti-CK118 (green) and anti-vimentin antibodies (red), followed by DAPI staining (blue). Isolated cultures of epithelial cells were only reacted with anti-CK18 antibody. BF: bright field image. Scale bar, 50 μm.

Supplementary Fig. S3 Preparation of human endometrial stromal cells.

In the process of dissociation of the uterine tissue, endometrial stromal cells were obtained. Endometrial stromal cells were fixed and double-stained with anti-CK18 (green) and anti-vimentin antibodies (red), followed by DAPI staining (blue). Isolated culture of stromal cells were only reacted with anti-vimentin antibody. Scale bar, 50 μm.

Supplementary Fig. S4 The distribution pattern of differentially methylated probes in endometrial epithelial cells.

(a) Venn graph showing the genomic distribution of DMRs. (b) Venn graph showing the CpG context distribution.

Supplementary Table S2 Annotation list of differentially methylated regions (DMRs).

Annotation by our Gene Ontology (GO) analysis showed that DMRs were enriched for cellular functions including chromosome, mitotic spindle, polyubiquitination, acetyl-CoA biosynthetic process, apoptotic signaling, and NIK/NF-kappaB signaling ($p<0.05$), suggesting that obesity affects certain cellular events in the presymptomatic uterus.

Supplementary Fig. S5 Pathway analysis of differentially methylated regions (DMRs).

DMRs are enriched for pyrimidine, EB virus, and B cell signaling pathways, and the methylation levels of genes are increased (hypermethylated, blue) or decreased (hypomethylated, red) in these three pathways.

Supplementary Table S3 Annotation list of enriched RefGenes among DMRs that shared between obese and stage I EC.

Annotation by our Gene Ontology (GO) analysis showed that shared DMRs were enriched for genes involved in chromatin binding, transcription factor binding, and neural development ($p<0.05$).

Supplementary Table S4 List of the shared RefGenes that were enriched in EB virus and B cell signaling pathways.

Shared DMRs are enriched for EB virus and B cell signaling pathways, and RefGenes in two pathways were listed.

Supplementary Figure S6 A model of endometrial cancer oncogenesis in obese women by a gradual decrease in methylation levels during persistent obesity.

Obesity induces a decrease in methylation levels of four RefGenes (POLR3A and HDAC1 in the EB virus infection pathway; ATF2 and EIF2AK4 in the B cell signaling) as shown in Fig.4. During persistent obesity, the methylation levels become progressively lower. Therefore, the signaling of aforementioned pathways will be impaired. This persistent impairment may contribute to the oncogenesis of EC.

Supplementary Figure S7 Comparison of the location of differentially methylated resions (DMRs) loci with SNPs.

To test the relationship of DMRs with DNA sequence variation, we compared the locations of CpG sites of shared RefSeqs (i.e., POLR3A and HDAC1 in the EB virus infection pathway; ATF2 and ELF2AK4 in the B cell signaling) with the location of SNPs. None of four DMRs are condensed within SNP clusters, suggesting that change of signals in the methylation array are unlikely to be due to DNA sequence variation, but rather due to altered methylation.

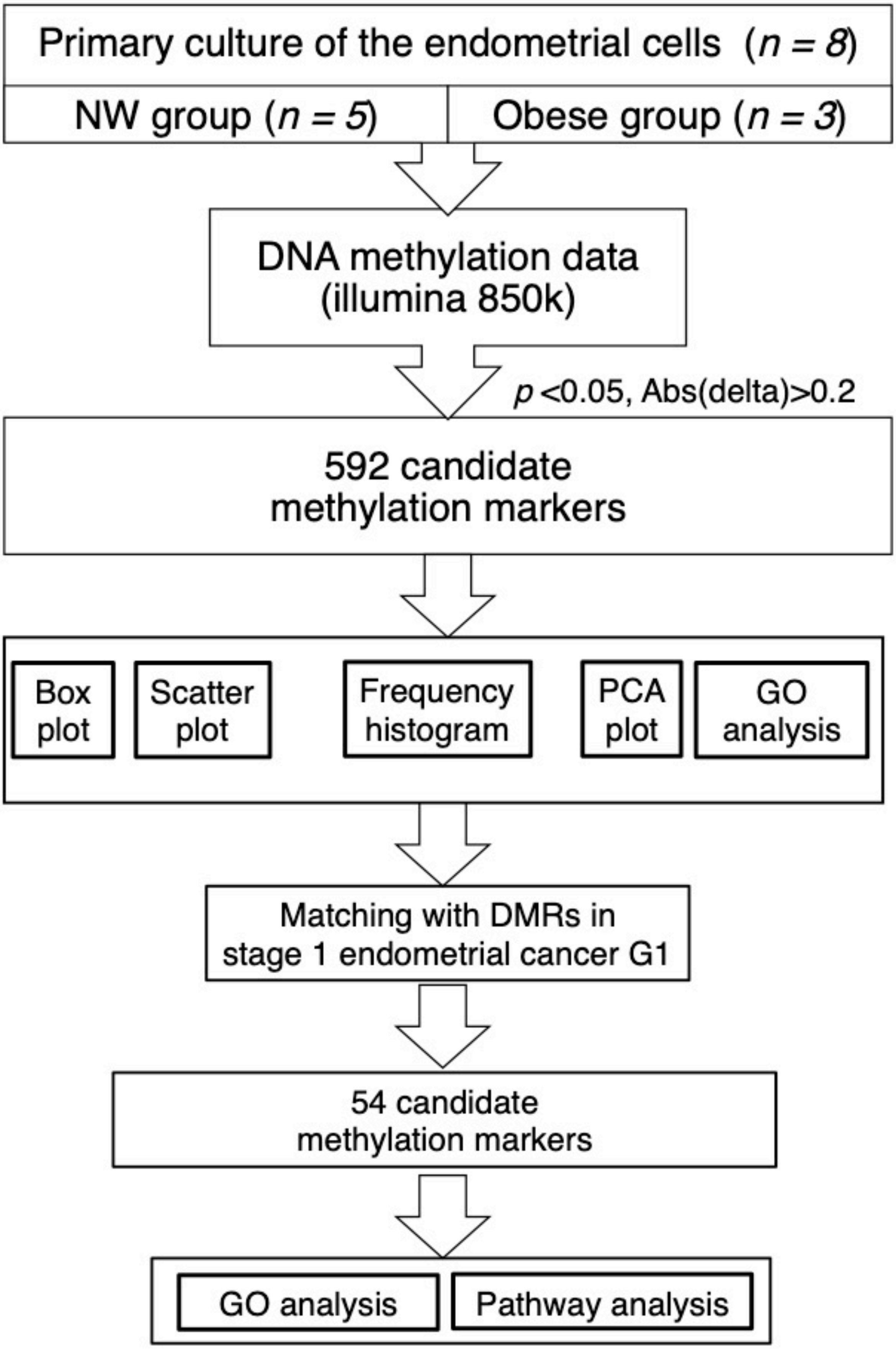

Supplementary Fig.S1

Table 1 - characteristics

No	1	2	3	4	5	6	7	8
	control					obesity		
Age(y)	49	41	45	49	44	47	46	26
BMI	17.9	18	18.6	20.9	23	31	32	43
gravidity	4	3	3	1	0	2	0	0
parity	3	3	3	1	0	2	0	0
day of menstrual cycle	38	6	20	30	87	8	20	55
Purity（%） of endometiral epithelial cells	81	80	85	83	87	81	89	93
Other s						Hyper tention	Hyper tention	Type2 diabetes Depression

Supplementary Table S1

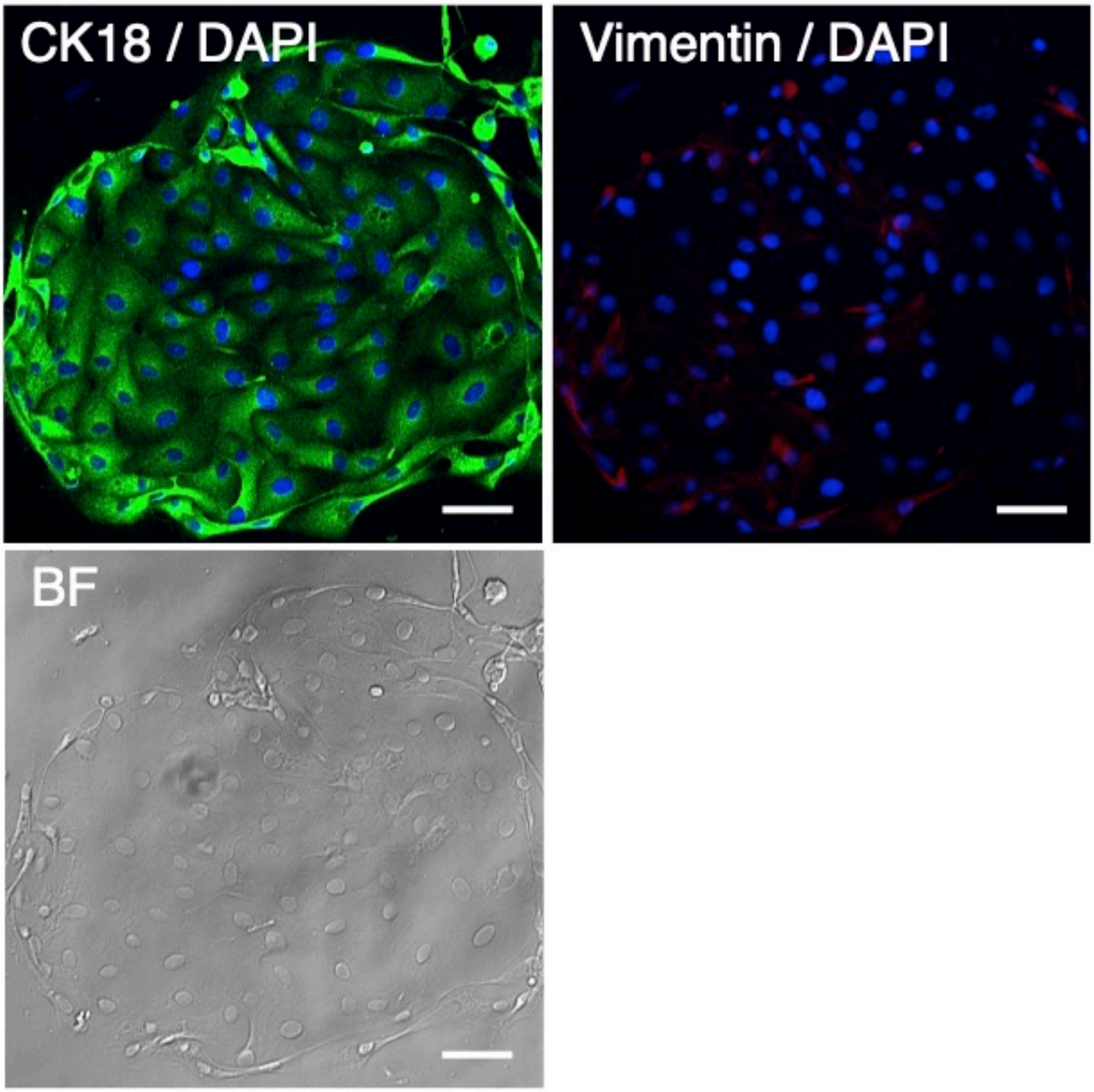

Supplementary Fig.S2

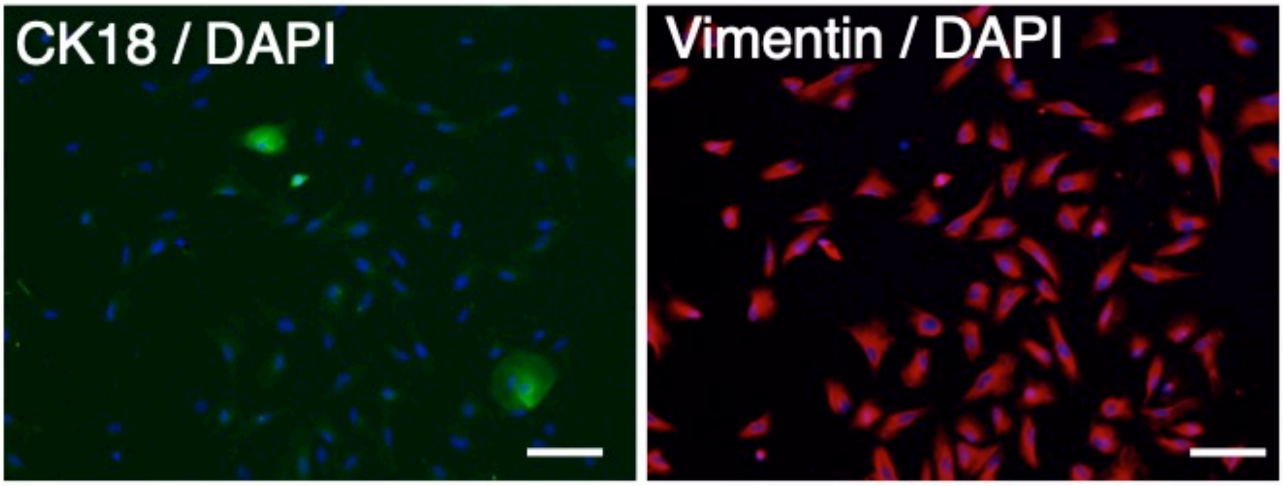

Supplementary Fig.S3

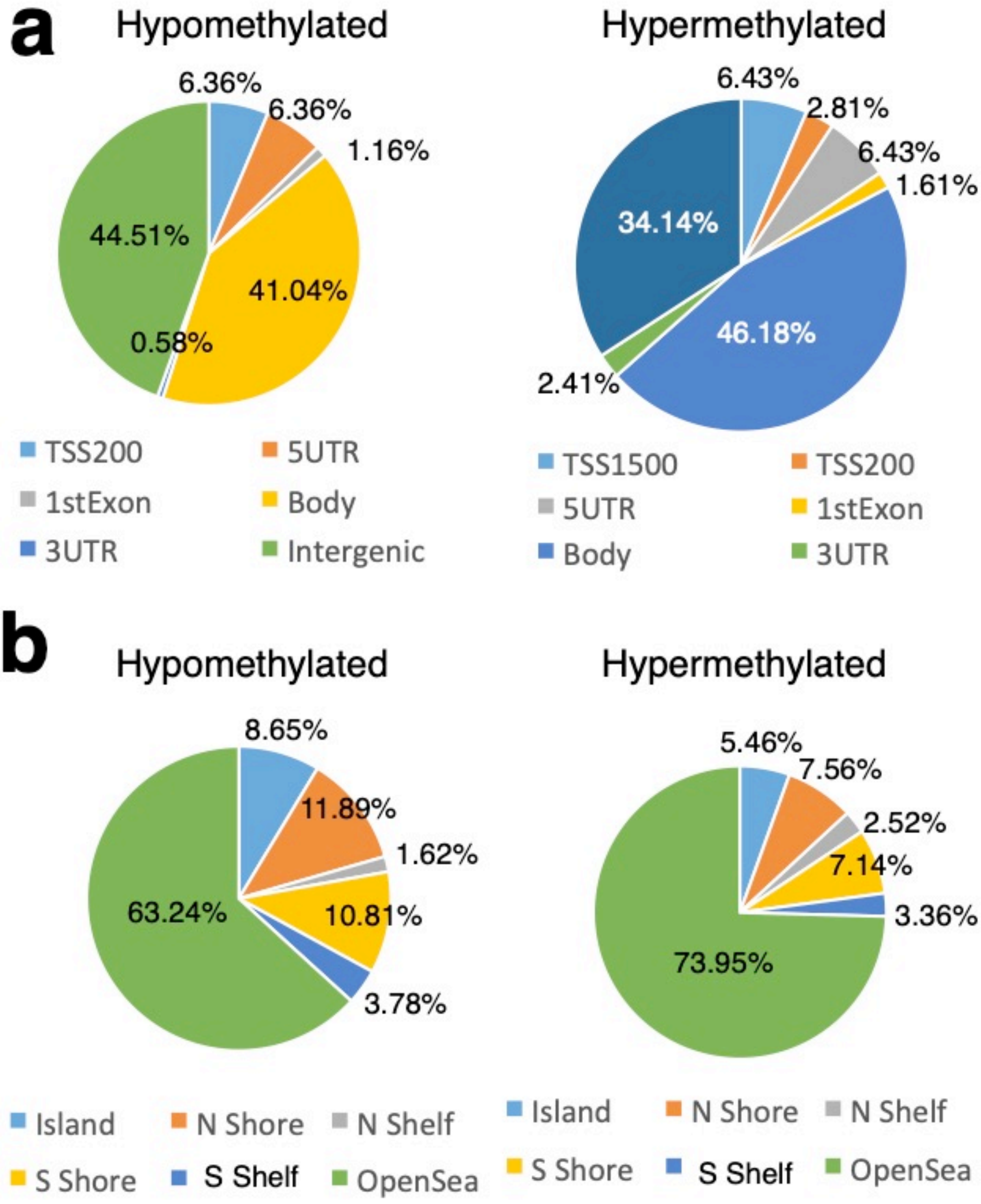

Supplementary Fig.S4

Annotation of DMRs

Category	Term	Count	%	P-Value
GOTERM_CC_DIRECT	chromosome, centromeric region	5	1.2	0.021
GOTERM_CC_DIRECT	mitotic spindle	4	1	0.04
GOTERM_CC_DIRECT	chromosome	6	1.5	0.046
GOTERM_BP_DIRECT	telomere maintenance via telomerase	4	1	0.0044
GOTERM_BP_DIRECT	establishment of protein localization to telomere	3	0.7	0.0051
GOTERM_BP_DIRECT	mitotic spindle assembly checkpoint	4	1	0.006
GOTERM_BP_DIRECT	cellular response to DNA damage stimulus	11	2.7	0.0063
GOTERM_BP_DIRECT	protein polyubiquitination	10	2.5	0.0084
GOTERM_BP_DIRECT	regulation of acetyl-CoA biosynthetic process from pyruvate	3	0.7	0.021
GOTERM_BP_DIRECT	intrinsic apoptotic signaling pathway in response to oxidative stress	3	0.7	0.032
GOTERM_BP_DIRECT	double-strand break repair	5	1.2	0.036
GOTERM_BP_DIRECT	NIK/NF-kappaB signaling	5	1.2	0.036
GOTERM_BP_DIRECT	response to lipopolysaccharide	8	2	0.036
GOTERM_BP_DIRECT	actin filament-based movement	3	0.7	0.04
GOTERM_BP_DIRECT	male meiosis I	3	0.7	0.045
GOTERM_BP_DIRECT	nervous system development	11	2.7	0.047
GOTERM_BP_DIRECT	osteoblast differentiation	6	1.5	0.047

Supplementary Table S2

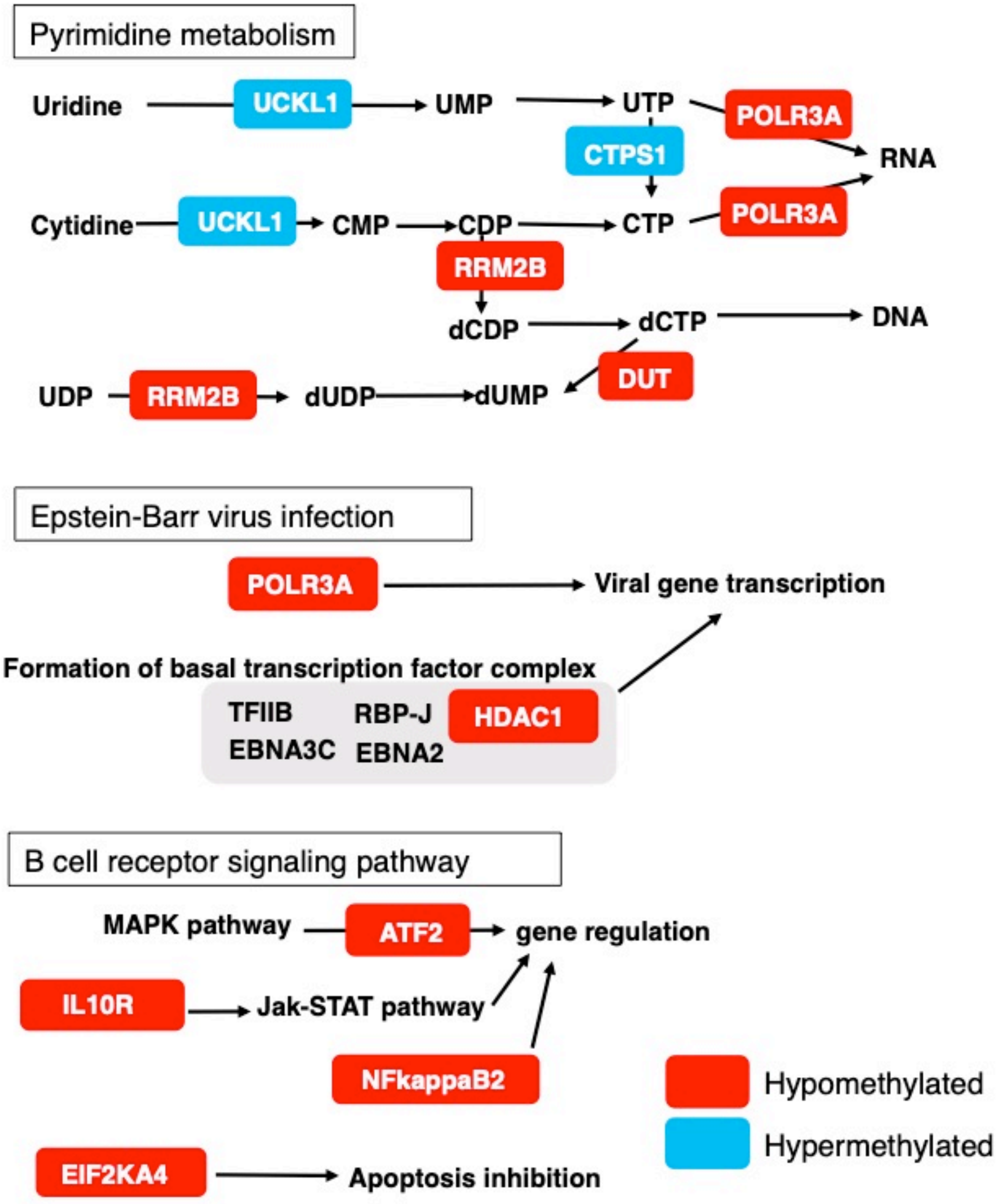

Supplementary Fig.S5

Annotation of DMRs

Category	Term	Count	%	PValue
GOTERM_CC_DIRECT	membrane	12	22.222	0.029267
GOTERM_MF_DIRECT	chromatin binding	6	11.111	0.003708
GOTERM_MF_DIRECT	transcription factor activity, RNA polymerase II distal enhancer sequence-specific binding	3	5.5556	0.013376
GOTERM_MF_DIRECT	Arp2/3 complex binding	2	3.7037	0.018515
GOTERM_MF_DIRECT	transcription factor binding	4	7.4074	0.039806
GOTERM_BP_DIRECT	nervous system development	4	7.4074	0.039119

Supplementary Table S3

Shared RefGenes enriched in EB virus and B cell signaling pathways

GENE-SYMBOL	Delta value of obese	LogRatio of β-value of obese	Delta value of Stage 1 EC	RefSeq ACCESSION	ENTREZ-GENEID	TYPE_OF_GENE	DESCRIPTION
POLR3A	-0.01086	-0.95504	-0.15931	NM_007055	11128	protein-coding	polymerase (RNA) III subunit A
HDAC1	-0.00768	-0.76564	-0.22362	NM_004964	3065	protein-coding	histone deacetylase 1
ATF2	-0.00536	-0.7483	-0.13971	NM_001880	1386	protein-coding	activating transcription factor 2
EIF2AK4	-0.00386	-0.63514	-0.22871	NM_001013703	440275	protein-coding	eukaryotic translation initiation factor 2 alpha kinase 4

Supplementary Table S4

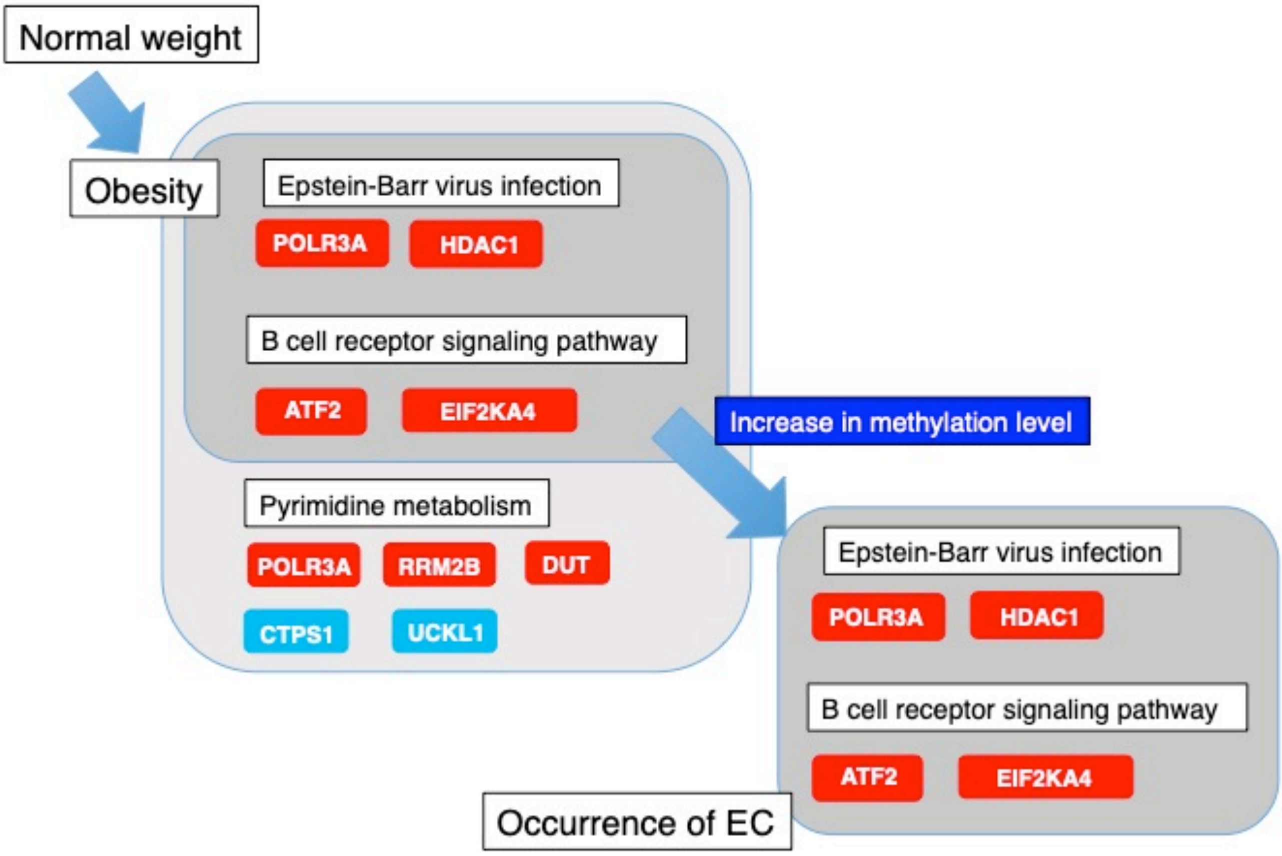

Supplementary Fig. S6

POLR3A

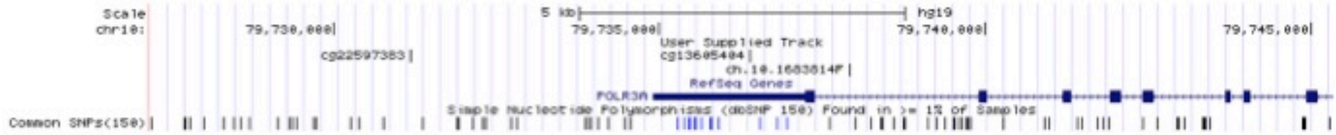

HDAC1

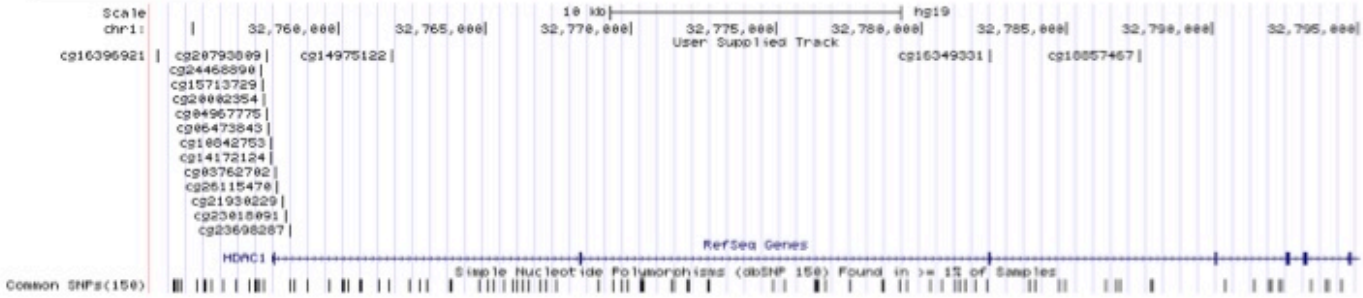

ATF2

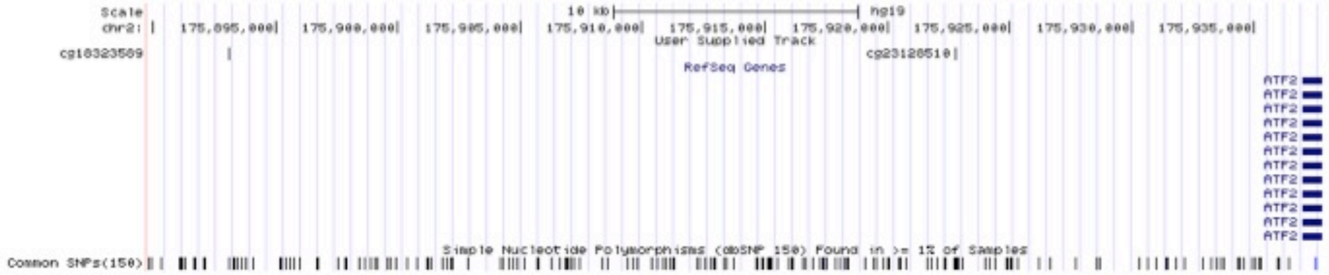

EIF2AK4

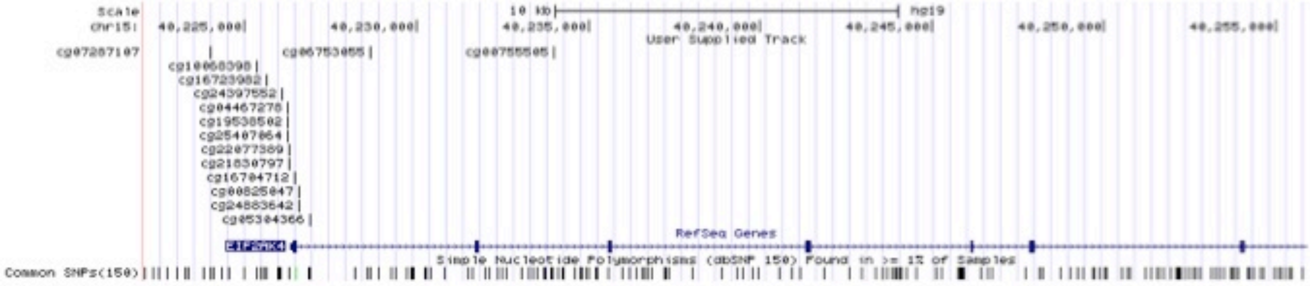

Supplementary Fig. S7
